# Targeted treatment of iron deficiency in prolonged critical illness: an opportunity to improve survival or not?

**DOI:** 10.1186/s13054-021-03590-w

**Published:** 2021-06-01

**Authors:** Jan Gunst, Greet Van den Berghe, Michael P. Casaer

**Affiliations:** Clinical Department and Laboratory of Intensive Care Medicine, Department of Cellular and Molecular Medicine, KU Leuven, Herestraat 49, 3000 Leuven, Belgium

**Dear editor,**

Lasocki et al. recently reported the results of the Hepcidane randomized controlled trial (RCT), randomizing long-stay intensive care unit (ICU) patients to hepcidin-guided treatment of iron deficiency versus standard care [1]. Although the intervention had no impact on the primary endpoint—post-ICU length of stay (LOS)—it apparently improved survival, a secondary endpoint. A subgroup analysis, comparing all control group patients with low hepcidin versus those in the intervention group with low hepcidin and effective supplementation (49%), confirmed a potential survival benefit. The authors should be commended for having performed this landmark RCT, investigating individualized therapy.

Translation of these findings into clinical practice, however, requires important information that is actually lacking. Indeed, the authors evaluated the effect of the intervention on the morbidity and mortality after ICU-discharge, hereby ignoring a potential effect occurring between randomization and discharge. Although patients were randomized shortly before ICU-discharge, the—unreported—time from randomization to discharge might be not negligible, since 12 patients died in this period (more in the intervention group). Likewise, in the Kaplan–Meier curves, the starting point is ICU-discharge (day 0), hereby excluding early deaths. The numbers at risk reported under these curves suggest that early deaths were equally excluded from the comparison of 90-day mortality rates. In addition, 3 patients who were exposed to the intervention were excluded because of previously unnoticed exclusion criteria, precluding intention-to-treat analysis. Could the authors report the hospital LOS, mortality rates and Kaplan–Meier curves from randomization onward in all randomized patients and in subgroup analysis?

Second, it appears as if the primary endpoint was unknown in a considerable number of patients. Indeed, patients with missing data were arbitrary assigned LOS of 90 days. Does the 75th percentile of LOS –90 days in both study groups– reflect missing primary outcome data in more than one quarter of patients? Could the authors report the number of missing primary outcome data and repeat the univariate comparison without imputation (although it is unclear if missing data occurred at random or not)?

Third, more than 1800 patients were excluded for unclear reasons. Could the authors specify the reasons, which would provide more insight in the included study population?

Finally, could the authors repeat the subgroup analysis independent of iron supplementation provided/omitted when indicated, as such is likely not a random phenomenon.

Undoubtedly, these additional data will allow to better appreciate the generalizability of the results of this important RCT.

## Data Availability

Not applicable.
