# Supplementary material for: Targeted treatment of iron deficiency in prolonged critical illness: an opportunity to improve survival or not?
Source: Crit Care. 2021 Jun 1;25:188. doi: 10.1186/s13054-021-03590-w (PMC8170975; doi:10.1186/s13054-021-03590-w)
Supplement: Related texts

# Reply: Authors’ response

**Authors:** Sigismond Lasocki

**Affiliations:** grid.7252.2 0000 0001 2248 3363 Département Anesthésie Réanimation, Université D’Angers, CHU Angers, 4 rue Larrey, 49933 Angers Cedex 9, France

## Yes, targeted treatment of iron deficiency may improve critically ill patients survival!

Gunst et al. raised several points regarding the results of our Hepcidane trial [1]. The first one concerns the delay between randomization and ICU discharge. Indeed, we initially planned to included patients about to be discharged (i.e. on the day discharge was decided) [2], but we faced logistical problem (the centralized measurement of hepcidin was available only on Thursdays) so patients had to be screened on Mondays and Tuesdays, to have their blood samples drawn on Wednesdays. This explains why some patients had complications and remained in the ICU after inclusion (12 (2.9%) died during this period). However, the delay between study inclusion and ICU discharge was equivalent in the two groups (medians [Q1–Q3], 2 [1–6] vs 2 [1–6] days), it is the same for the post-randomization length of stay (36 [15–90] vs 36 [15–90] days). Three patients had to be excluded after randomization for unnoticed exclusion criteria (2 in control and 1 in intervention arm, respectively) and 1 patient withdrew his consent in the intervention group. These patients were therefore not followed up (2 in each group, see flowchart in Fig. 1 of the article). As indicated in the Table 2 of the manuscript, in intention-to-treat analysis (*n* = 399 patients, taking into account the patients dead in the ICU) the death rate at D90 was lower in the intervention than in the control arm (16 (8.0%) vs 33 (16.7%) deaths, *p* = 0.008). This mortality rate was also lower in patients with iron deficiency (ID) treated in the intervention arm compared to those with ID not treated patients in the control arm (subgroup analysis) at D90 (3 (5.45%) deaths among 55 patients vs 20 (19.05%) among 105 patients, *p* = 0.02). The Kaplan Meier survival curves from inclusion till D90 (as initially scheduled in the statistical plan) and till D360 (as requested by reviewers) are reported in the figure.

**Fig. 1:** Kaplan–Meier survival curves till D90 (upper panels) and D360 (lower panels) after inclusion. Panels on the left (**a** & **c**) Intention-to-treat analysis (all population). Panels on the right (**b** & **d**) ID treated vs ID non treated patients (Scheduled subgroup analysis on patients with hepcidin < 41 μg/L treated in the intervention arm and not treated in the control arm)

As indicated in the flowchart of the study, the follow-up data for length of stay was unknown for only 11 patients, i.e. 2.8% of the study population. A sensitivity analysis excluding these patients did not find any difference between the two study arms.

We do not know the reason for the non-inclusion of these 1800 patients; however, it is mainly related to the inclusion window limited at 2 days in the week, and also to competing studies: Patients were eligible only at the end of their ICU stay, and many of them were already included in other studies, preventing their inclusion in the Hepcidane trial.

Finally, we compared patients with ID treated or not [2], but it does not make much sense to compare patients with ID (treated or not in the intervention arm) to patients with ID not treated, since it is the treatment of ID that is supposed to be beneficial.

We hope that we have answered the questions, but we fully agree that our study provides only some signal in favor of the treatment of ID based on hepcidin quantification, which need to be confirmed.
